# Decrease in Irisin in Patients with Chronic Kidney Disease

**DOI:** 10.1371/journal.pone.0064025

**Published:** 2013-05-07

**Authors:** Ming-Shien Wen, Chao-Yung Wang, Shuei-Liong Lin, Kuo-Chun Hung

**Affiliations:** 1 Department of Cardiology, Chang Gung Memorial Hospital, and Chang Gung University College of Medicine, Taoyuan, Taiwan; 2 Renal Division, Department of Medicine, National Taiwan University Hospital, Taipei, Taiwan; 3 Graduate Institute of Physiology, National Taiwan University, College of Medicine, Taipei, Taiwan; Virginia Commonwealth University, United States of America

## Abstract

Patients with chronic kidney disease have abnormal energy expenditure and metabolism. The mechanisms underlying altered energy expenditure in uremia are unknown and remain to be elucidated. Irisin is a peroxisome proliferator-activated receptor γ coactivator 1-α–dependent myokine, and it increases energy expenditure in the absence of changes in food intake or activity. We hypothesize that chronic kidney disease patients have altered irisin levels. We measured resting irisin levels in 38 patients with stage 5 chronic kidney disease and in 19 age- and sex-matched normal subjects. Plasma irisin levels were significantly decreased in chronic kidney disease patients (58.59%; 95% CI 47.9%–69.2%, *p*<0.0001). The decrease in irisin levels was inversely correlated with the levels of blood urea nitrogen and creatinine. Further association analysis revealed that irisin level is independently associated with high-density lipoprotein cholesterol level. Our results suggest that chronic kidney disease patients have lower than normal irisin levels at rest. Furthermore, irisin may play a major role in affecting high-density lipoprotein cholesterol levels and abnormal energy expenditure in chronic kidney disease patients.

## Introduction

Patients with chronic kidney disease (CKD) have altered energy expenditure.[1] Epidemiological studies have demonstrated that the prevalence of metabolic imbalance and abnormal energy homeostasis in patients with CKD ranges from 20% to 80%.[2]–[4] Altered energy expenditure in CKD results in weight gain, obesity, protein-energy waste, and higher mortality.[5] The mechanisms underlying deregulation of energy expenditure in CKD patients are multifactorial. The kidneys account for approximately 7% of resting energy expenditure.[6] Additionally, decreased renal blood flow and loss of renal function are associated with lower renal oxygen consumption and hypometabolic status.[7] Kidney failure results in multiple abnormalities in cellular metabolism, including impaired glucose metabolism, altered cellular protein turnover, metabolic acidosis, and inflammation.[8] Moreover, an elevated inflammatory response, uncontrolled diabetes, and protein catabolism can contribute to increased energy expenditure. Lower physical activity, impaired skeletal muscle metabolism and insulin resistance can lead to decrease energy expenditure.[6], [9], [10]

Although it is well-documented that energy expenditure is altered in CKD, the results of related clinical studies are controversial. Different studies have reported the resting energy expenditure of patients with CKD to be similar to,[2] higher than,[7] or lower than[4], [11] that of healthy individuals. These divergent results can probably be explained by the different study designs and the patient population studied.[12] CKD encompasses various disease entities, and patients receive different treatments and kidney replacement therapies. Unless the underlying mechanisms are clear, clinical studies cannot easily determine the relationship between kidney failure and energy metabolism.

Irisin has been identified recently as an exercise-induced hormone secreted by skeletal muscle. Boström et al. observed that muscle-specific overexpression of the peroxisome proliferator-activated receptor γ (PPARγ) coactivator 1α (PGC-1α) in mice induced a brown fat-like gene program.[13] Mice overexpressing PGC-1α are not only resistant to obesity but also prone to the formation of multilocular and UCP1-positive adipocytes.[14] Profiling of the muscle genes activated by PGC-1α revealed fibronectin type III domain containing 5 (FNDC5), which undergoes proteolytic cleavage to release irisin into the blood. Irisin can activate oxygen consumption and thermogenesis in white fat cells. In that same study, the injection of irisin into mice increased total body energy expenditure and reduced obesity in mice fed a high-fat diet. The discovery of the PGC-1α–FNDC5–irisin axis has broad implications for metabolism and energy homeostasis.[15] However, the role of irisin in humans and in clinical disease remains unknown. We hypothesize that patients with CKD may have altered irisin levels and that this may be responsible for the deregulated energy expenditure due to uremia.

## Materials and Methods

### Subjects

This study enrolled 38 consecutive patients with stage 5 CKD who did not undergo kidney replacement therapy (52.6% female; mean age, 57.4±2.5 years) from the outpatient clinics of Chang Gung Memorial Hospital from 2008 to 2011. The diagnosis and staging of CKD were based on the National Kidney Foundation Disease Outcomes Quality Initiative (KDOQI) criteria. Patients with diabetes, impaired liver function (bilirubin >1.6 mg/dL), uncontrolled hypertension, heart failure, cerebrovascular disease, neurodegenerative disorder, systemic lupus erythematosus, severe obesity (body mass index [BMI] >35), severe hyperlipidemia (total cholesterol >300 mg/dL; triglyceride >400 mg/dL), or those currently taking immunosuppressive agents were excluded from this study. Nineteen sex- and age-matched healthy volunteers (mean age, 59.3±1.8 years) were used as controls. These healthy volunteers were asked to complete detailed clinical questionnaires and undergo blood tests to exclude renal function impairment, diabetes, abnormal liver function, heart failure, and severe obesity (BMI >35). Subjects who exercised regularly or strenuously within 1 month of the study were also excluded. All subjects provided written informed consent. The protocol was approved by the institutional review boards of Chang Gung Memorial Hospital and it adhered to the Helsinki Declaration.

### Measurement of irisin

Blood samples were taken and questionnaires were completed between 6:00 AM and 9:00 AM, after 12 h of fasting. Plasma was prepared from a 10-mL blood sample. A volume of 35 μL of plasma was precleared for albumin and IgG with a ProteoExtract Kit (Calbiochem, Billerica, MA, USA). The samples were then concentrated with an Ultra-2 Centrifugal Filter (Amicon, Billerica, MA, USA) to a volume of approximately 100 µL, with a concentration of >6 µg/µL. Protein samples (150 µg) were deglycosylated with PNGase F (New England Biolabs, Ipswich, MA, USA), and the samples were made up to a concentration of 2 µg/µL and boiled with 1× sample buffer with a reducing agent. The proteins were then resolved by 12% sodium dodecyl sulfate polyacrylamide gel electrophoresis (SDS-PAGE) and transferred onto a polyvinylidene fluoride (PVDF) membrane (Millipore, Billerica, MA, USA). Next, membranes were blocked in 5% milk PBST (0.1% Tween 20 in PBS) and incubated with rabbit anti-FNDC5 (Irisin) polyclonal antibody (Abcam, Cambridge, MA, USA). Bands were visualized with enhanced chemiluminescence (ECL) reagents (Amersham Biosciences, Piscataway, NJ, USA) and analyzed with Image J software (National Institutes of Health, Bethesda, MD, USA). Finally, blots from different patients were normalized using protein lysates from 293T cells overexpressing FNDC5 protein.

Plasma irisin concentrations were also measured with a commercially available enzyme immunoassay kit (Phoenix Pharmaceuticals, Burlingame, CA, USA). [16]

### Cell culture and treatment

Primary human skeletal muscle (SkMC) cells were obtained from Lonza (Walkersville, MD, USA). SkMC cells were cultured in skeletal muscle growth medium (SkGM^TM^) with human epidermal growth factor, fetuin, bovine serum albumin, dexamethasone, and insulin according to the manufacturer’s instructions. Mouse-derived C2C12 myoblasts were maintained in Dulbecco’s modified Eagle’s medium supplemented with 10% fetal bovine serum. The differentiated myotubes were prepared according to a previously described protocol. [17] SkMC or C2C12 cells were synchronized under serum-free conditions overnight before treatment with indoxyl sulfate or hydrogen peroxide (Sigma, St. Louis, MO, USA).

### Immunoblot analysis

Protein extracts were prepared in cell lysis buffer (Cell Signaling, Beverly, MA, USA). Equal amounts of protein extracts were resolved by in an SDS-PAGE gel and transferred onto PVDF membranes. Membranes were blocked for 1 h and probed with primary antibodies overnight. The primary antibodies used were FNDC5, PGC-1α (clone 4C1.3, Calbiochem), and β-actin (Sigma). Membranes were then probed with a secondary antibody and detected with an ECL reagent (PerkinElmer, Walthem, MA, USA). The final protein optical densities were normalized with β-actin bands.

### Statistical methods and analyses

Continuous variables for normal distribution were tested with the Kolmogorov–Smirnov test. Data with a normal distribution (expressed as mean ± SD) were analyzed using Student’s *t* test. We used Pearson’s correlation coefficient to calculate the correlation between irisin level and parametric continuous variables, and Spearman’s correlation coefficient to calculate the nonparametric variables. Because irisin levels and lipid profiles are influenced by renal function, the association between irisin and lipid profiles was calculated in this study by using partial correlation coefficients, which were adjusted for creatinine. All reported *p* values were 2-sided, and a *p* value of <0.05 was considered statistically significant. Statistical analyses were performed with SPSS software (IBM).

## Results

Table 1 summarizes the characteristics of the study patients. Patients with CKD had higher blood urea nitrogen (BUN) and creatinine levels and blood pressure, and lower hemoglobin and high-density lipoprotein (HDL) cholesterol levels than did normal controls. Additionally, because this study excluded patients with diabetes mellitus and severe hyperlipidemia, CKD patients did not significantly differ from normal subjects in terms of fasting glucose, high-sensitivity C-reactive protein (hs-CRP), total cholesterol, triglyceride, low-density lipoprotein (LDL) cholesterol, and uric acid levels.

**Table 1:** Demographic and clinical characteristics of normal controls and patients with chronic kidney disease.

	Normal (n = 19)	CKD (n = 38)
Age	59.3 (1.8)	57.4 (2.5)
Sex (female)	10 (52.6%)	20 (52.6%)
Weight (kg)	62.2 (2.2)	63.5 (2.0)
Height (cm)	161.9 (1.6)	159.1 (1.4)
Body mass index (kg/m^2^)	23.6 (0.7)	25.1 (0.8)
Waist circumferences (cm)	82.3 (2.1)	89·0 (1.2)
Fasting glucose (mg/dL)	99.5 (3.1)	97.6 (2.1)
Hypertension	0 (0%)	22 (60.0%)
Smoking	5 (26%)	4 (10.5%)
BUN (mg/dL)	12.4 (0.7)	67.9 (7.3)†
Creatinine (mg/dL)	0.8 (0.0)	6.4 (0.8) †
Total cholesterol (mg/dL)	195.2 (6.2)	206.6 (6.7)
Triglyceride (mg/dL)	114.6 (18.1)	177.3 (15.4)
LDL(mg/dL)	118.5 (4.1)	124.9 (7.2)
HDL(mg/dL)	55.1 (4.3)	39.4 (3.9) †
Uric acid (mg/dL)	6.1 (0.4)	7.6 (0.3)
hs-CRP	1.9 (0.6)	2.7 (0.4)
Hemoglobin (g/L)	13.5 (0.5)	11.1 (0.3) †
Patients receiving (drug class)		
ACE inhibitors/ARBs	–	11
β blockers	–	8
Calcium channel blockers	–	13
Diuretics	–	7

Data are represented as n (n%) or mean (standard error of the mean), unless otherwise specified. *ACE*, angiotensin-converting enzyme; *ARB*, angiotensin receptor antagonist; *BUN*, blood urea nitrogen; *CKD*, patients with chronic kidney disease; *HDL*, high-density lipoprotein cholesterol; *hs*-*CRP*, high-sensitivity C-reactive protein; *LDL*, low-density lipoprotein cholesterol.

† indicates significant differences between CKD patients and normal controls, with p<0.05.

**Figure 1:**
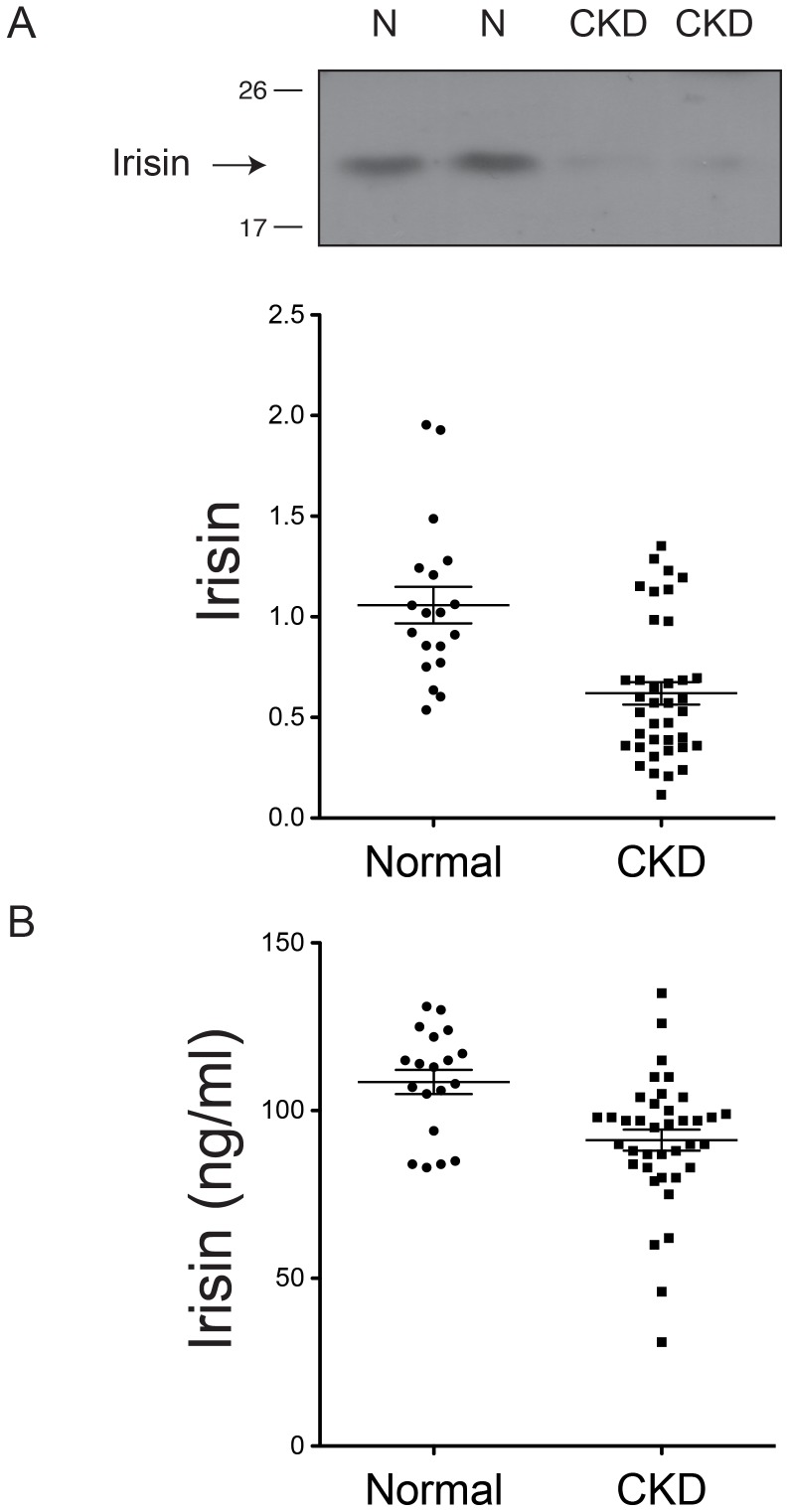
Irisin in healthy subjects and in patients with chronic kidney disease. A, Irisin expression measured by western blot analysis in normal control subjects (N) (n = 19) and in chronic kidney disease (CKD) patients (n = 38). Representative blots were chosen from 2 healthy subjects and 2 CKD patients. B, Irisin expression measured by ELISA in normal control subjects and CKD patients.

After albumin/IgG depletion and deglycosylation, we were able to detect irisin with western blotting, according to the protocol described previously (Fig. 1*A*).[13] An immunoreactive band at 22 kDa was obtained for serum irisin and positive control protein lysates. Additionally, variations in the plasma irisin levels were observed among the 19 normal subjects after normalization with the control protein lysate (1.058±0.091; 95% confidence interval [CI] 0.8677–1.248). The minimum irisin level was 0.54 and the maximum level was 1.954, although this study excluded subjects who had participated in regular and strenuous exercise within 1 month of the study. Compared with normal subjects, the irisin levels in CKD patients were lower (0.6199±0.056; 95% CI 0.5072–0.7326) and significantly decreased (58.59%; 95% CI 47.9%–69.2%, *p*<0.0001) (Fig. 1*A*).

To reconfirm the findings from western blotting, we examined the plasma irisin levels with an irisin/FNDC-5 (extracellular domain molecule: epitope 16-127) assay kit.[16] The normal subjects had a mean plasma irisin concentration of 108.5±3.6 ng/mL (range, 83.0– 131.0 ng/mL). The mean irisin levels in CKD patients were 91.2±3.1 ng/mL (range, 31.0–135.5 ng/mL). The irisin levels in CKD patients measured with the irisin assay kit were decreased when compared with the levels in normal subjects (84.07%; 95% CI 78.2%–89.9%, p = 0.0014) (Fig. 1*B* *)*. The result from the irisin assay kit is consistent with the findings from western blotting. However, the degree of decrease detected by the irisin assay kit is less than that by western blotting.

In this study, an association analysis was performed to determine the correlation coefficient of plasma irisin level with fasting glucose, hemoglobin, total cholesterol, triglyceride, LDL cholesterol, HDL cholesterol, hs-CRP, uric acid, and hemoglobin levels; BMI; or smoking (Table 2). Among these factors, BUN and creatinine were both negatively associated with the level of irisin. In contrast, HDL cholesterol and hemoglobin levels were positively associated with irisin levels. Because CKD is often associated with lower hemoglobin and HDL cholesterol levels,[18], [19] the positive association between HDL cholesterol and hemoglobin levels, and irisin may be due to impaired renal function rather than irisin alone. Therefore, in this study, a partial correlation coefficient analysis adjusted for creatinine was performed to determine whether HDL cholesterol or hemoglobin is independently associated with irisin. After adjusting for renal function, no correlation was observed between hemoglobin and irisin. Interestingly, HDL cholesterol was found to have a significant positive correlation with irisin (coefficient 0.460; *p* = 0.008) (Table 3). These findings suggest that irisin levels are decreased in CKD patients and independently associated with HDL cholesterol levels.

**Table 2:** Correlation coefficient between irisin and serum biochemistry findings.

	Irisin	
Covariate	Coefficient	p Value(2-tail)
Total Cholesterol	−0.085	0.534
Triglyceride	−0.176	0.191
LDL	−0.104	0.518
HDL	0.449	0.001†
Uric acid	0.051	0.713
Glucose	−0.114	0.414
BUN	−0.368	0.005†
Creatinine	−0.410	0.002†
Hemoglobin	0.364	0.005†
hs-CRP	0.098	0.537
Height	0.177	0.189
Weight	0.007	0.956
Body mass index	−0.092	0.495
Smoking	0.100	0.458

*BUN*, blood urea nitrogen; *HDL*, high-density lipoprotein cholesterol; *hs-CRP*, high-sensitivity C-reactive protein; *LDL*, low-density lipoprotein cholesterol.

**Table 3:** Partial correlation coefficient between irisin and serum biochemistry findings, adjusted for creatinine.

	Irisin	
Covariate	Coefficient	p Value(2-tail)
Total Cholesterol	−0.110	0.550
Triglyceride	−0.332	0.063
LDL	−0.125	0.496
HDL	0.460	0.008†
Uric acid	0.150	0.414
Glucose	−0.128	0.486
Hemoglobin	0.279	0.121
hs-CRP	0.008	0.964

*BUN*, blood urea nitrogen; *HDL*, high-density lipoprotein cholesterol; *hs-CRP*, high-sensitivity C-reactive protein; *LDL*, low-density lipoprotein cholesterol.

**Figure 2:**
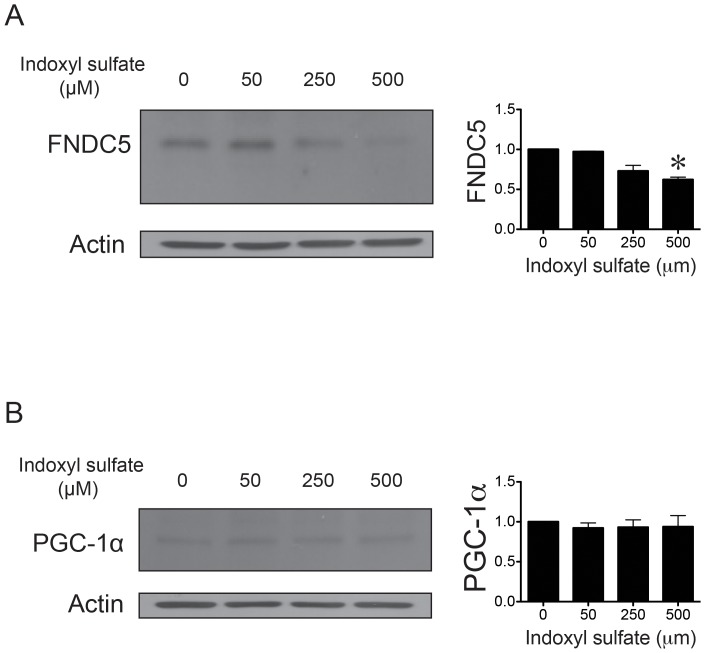
Indoxyl sulfate modulates FNDC5 expression in skeletal muscle cells. A, Dose-response relationship for the decrease in FNDC5 expression in skeletal muscle cells treated with indoxyl sulfate for 24 h. (n = 3; *, p<0.05) B, PGC-1α expression analysis by western blot of skeletal muscle cells treated with indoxyl sulfate for 24 h.

We next tested whether uremic toxin has a direct effect on the expression of irisin in skeletal muscle cells. Indoxyl sulfate, which is a protein-bound uremic toxin, increases significantly during kidney function deterioration.[20] Recent studies showed that the levels of indoxyl sulfate are associated with kidney disease progression and mortality in CKD patients. [21], [22] We treated human skeletal muscle cells with indoxyl sulfate at different concentrations (0, 50, 250 and 500 µM) for 24 h. In these cells, FNDC5 protein levels decreased in a dose-dependent manner after 24 h treatment of indoxyl sulfate. (Fig. 2*A*) The PGC1-α protein levels were not altered by indoxyl sulfate. (Fig. 2*B*) The irisin released into the culture medium of human skeletal muscle cells or differentiated C2C12 myoblasts, measured by the irisin assay kit after 24 h treatment of 500 µM indoxyl sulfate, were also significantly decreased when compared with controls (36.3±2.3 ng/mL vs 57.8±2.1 ng/mL, *p* = 0.01).

## Discussion

This study revealed that irisin levels are decreased in CKD patients without diabetes. Lower levels of irisin are independently associated with lower HDL cholesterol levels. These findings suggest that irisin may be involved in the regulation of HDL cholesterol levels.

These findings have several clinical implications. Exercise is known to decrease inflammation and oxidative stress and improve muscular strength and the quality of life in CKD patients. Further studies are needed to determine whether irisin have effects similar to those induced by exercise in patients with CKD. CKD patients without diabetes have impaired glucose oxidation and tissue insulin sensitivity due to uremia.[23], [24] Insulin resistance and concomitant hyperinsulinemia play an important role in cardiovascular morbidity and mortality in patients with CKD. The exact cause of insulin resistance in CKD patients without diabetes remains unknown. Several studies have suggested that the insulin resistance might be due to acidosis, inflammation, and uremia.[24] It is not known whether decreased irisin levels lead to insulin resistance in these patients. However, the fact that irisin improves glucose homeostasis and insulin resistance clearly indicates the therapeutic potential of irisin for insulin resistance in patients with CKD.[13] The efficacy of irisin in improving the outcome of CKD patients with cardiovascular disease is of worth investigating.

The correlation between serum HDL cholesterol and plasma irisin is intriguing. Direct mechanistic evidence for the effect of irisin on HDL cholesterol metabolism is lacking. Previous studies have established that exercise alone can increase HDL cholesterol levels; [25]–[27] however, further studies with irisin knockout mice would be required to determine if irisin is solely responsible for this increase. Nevertheless, the fact that higher irisin levels are associated with higher HDL cholesterol levels can be exploited for its therapeutic potential. HDL cholesterol protects against atherosclerosis through the reverse transport of cholesterol and its anti-inflammatory activity.[28] Clinical trials have indicated that increased HDL cholesterol levels coupled with niacin therapy are associated with a lower incidence of myocardial infarction and a reduction in long-term mortality.[29] Additionally, HDL cholesterol concentration is a strong inverse predictor of vascular events in the general population.[30] Many pharmacotherapeutic strategies (e.g., direct augmentation of apolipoprotein A−I, inhibition of cholesteryl ester transfer protein, activation of the high-affinity niacin receptor, and activation of the liver X receptor) target HDL cholesterol in an effort to reduce the remaining cardiovascular disease burden, despite the currently available optimal medical therapies.[31] The ability of irisin to directly increase HDL cholesterol and affect the atherosclerosis process requires further study.

The mechanism underlying the decrease in irisin in CKD is unknown. Our results showed that indoxyl sulfate decreases FNDC5 expression in skeletal muscle cells and irisin level in the cell culture medium. The effects of indoxyl sulfate on FNDC5 and irisin levels are not due to alterations of PGC1-α protein levels. We believe that this finding provides direct evidence on how uremia affects irisin level. However, the effects of indoxyl sulfate could only provide partial explanations about the decreased irisin levels in CKD patients. Our current knowledge about irisin is limited. For example, there are currently no data on the protein-binding rate, metabolic pathway, and excretion route of irisin. Further studies on the comprehensive metabolic pathway of irisin will be needed to obtain a whole picture of the role of irisin in CKD patients. Another possibility is that CKD patients may have lower muscle volume. Irisin is produced within muscle, and total muscle volume can affect the irisin level. It will be interesting to test in the future whether total muscle volume or exercise has a substantial impact on irisin levels. Patients with CKD may be less physically active than normal subjects, which may result in a lower irisin level. This study used questionnaires to exclude subjects who claimed to have exercised strenuously or regularly within 1 month before to the study. Resting energy expenditure measurements and 24-h activity monitoring will clarify this possibility. Moreover, it will be useful to test irisin levels both before and after exercise training.

We have examined the irisin levels with both western blotting and an enzyme immunoassay kit, and confirmed the decreased levels of irisin in CKD patients. However, we noticed a discrepancy between the irisin levels detected by western blotting and enzyme immunoassay. The plasma samples used for these 2 different detection methods were processed differently. Plasma samples used for enzyme immunoassay were unprocessed, whereas those used for western blotting detection were deglycosylated and precleared of IgG and albumin. We believe these different sample preparation methods may have resulted in the discrepancy in irisin levels. The irisin levels detected with western blotting are more likely to be of the free form unbound to proteins. Future work on the protein-binding ratio of irisin and irisin metabolism would be needed to resolve these questions.

This study has some limitations. Our strict patient selection criteria excluded patients with diabetes, liver function abnormalities, heart failure, stroke, systemic lupus erythematosus, and severe obesity. Therefore, the results of this study are not applicable to all CKD patients. Further, this study did not measure the subfractions of HDL cholesterol. Different subfractions of HDL cholesterol may affect cardiovascular outcomes differently.[18] The effect of irisin on HDL cholesterol composition warrants further study. Moreover, our study design did not allow us to infer the mechanism of action of irisin. Further therapeutic clinical trials or animal studies are necessary to clarify the mechanisms underlying the effects of irisin. It is also important to test if the changes in irisin levels correlate with the progression of CKD from stage 1 to stage 5.

In summary, this study demonstrated that irisin levels are decreased in patients with CKD and are positively associated with HDL cholesterol concentration. Irisin may be a promising therapeutic agent for treating cardiovascular disease in CKD patients.
